# Supplementary material for: An Aqueous Process for Preparing Flexible Transparent Electrodes Using Non-Oxidized Graphene/Single-Walled Carbon Nanotube Hybrid Solution
Source: Nanomaterials (Basel). 2023 Aug 3;13(15):2249. doi: 10.3390/nano13152249 (PMC10421273; doi:10.3390/nano13152249)
Supplement: Supplementary file 1 [file nanomaterials-13-02249-s001.zip › nanomaterials-2486234-supplementary.pdf]

SUPPLEMENTARY INFORMATION

An aqueous process to flexible transparent electrodes using non-oxidized graphene and SWNT hybrid solution

Min Jae Oh,[1,†] Gi-Cheol Son,[2,†] Minkook kim,[1] Junyoung Jeon,[1] Yong Hyun Kim,[1] Myungwoo Son[1,*]

[1]Artificial Intelligence & Energy Research Center, Korea Photonics Technology Institute (KOPTI), Gwangju 61007, Republic of Korea

[2]School of Materials Science and Engineering, Gwangju Institute of Science & Technology (GIST), Gwangju 61005, Republic of Korea

*e-mail: mwson@kopti.re.kr

Keywords: flexible transparent electrode, non-oxidized graphene, single-walled carbon nanotube, aqueous solution, transparent thin film transistor

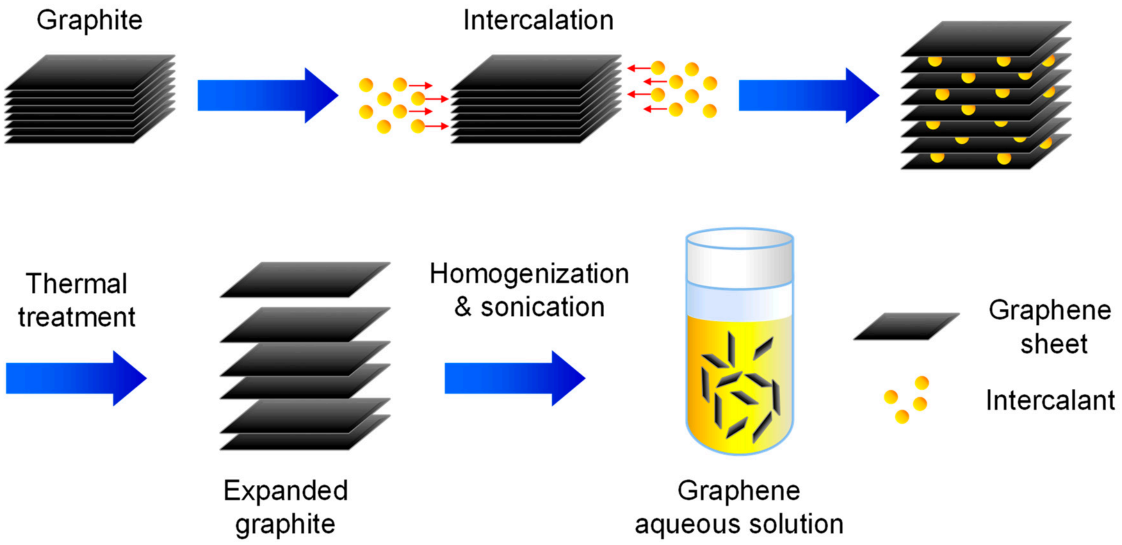

Figure S1. Schematic illustration showing the sequential procedures for preparing non-oxidized graphene aqueous solution using halogen intercalation method.

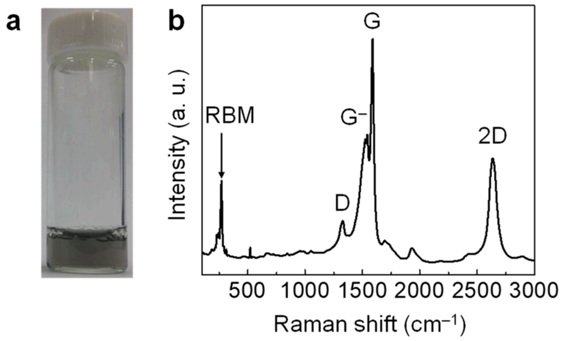

Figure S2. (a) Photograph of aqueous SWNT solution and (b) Raman spectrum of SWNT film.

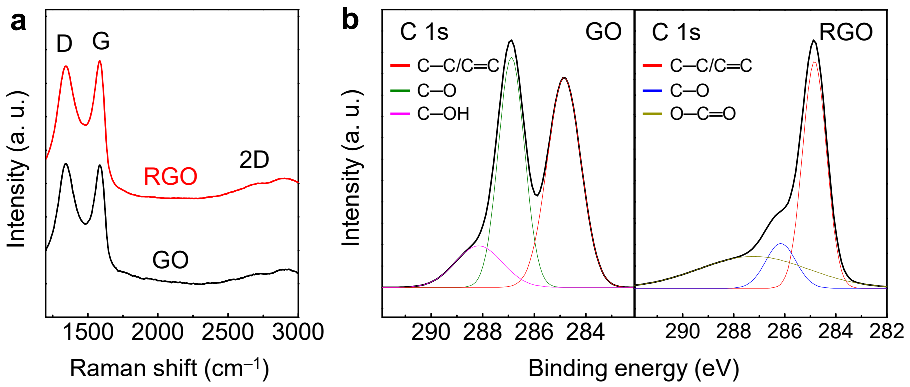

Figure S3. (a) Raman spectra and (b) XPS spectra of GO and RGO synthesized by Hummers' method.

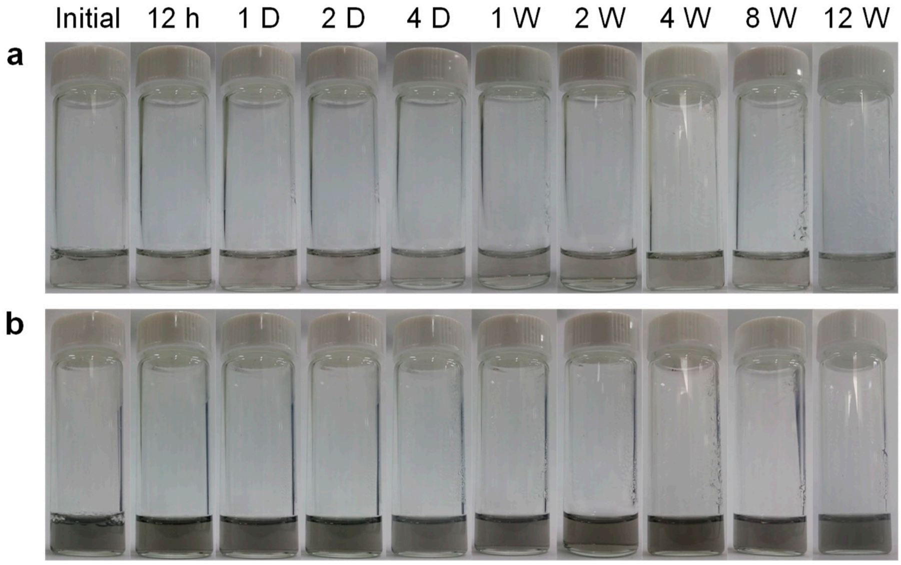

Figure S4. Photographs of (a) non-oxidized graphene and (b) aqueous SWNT solutions showing the dispersion stability.

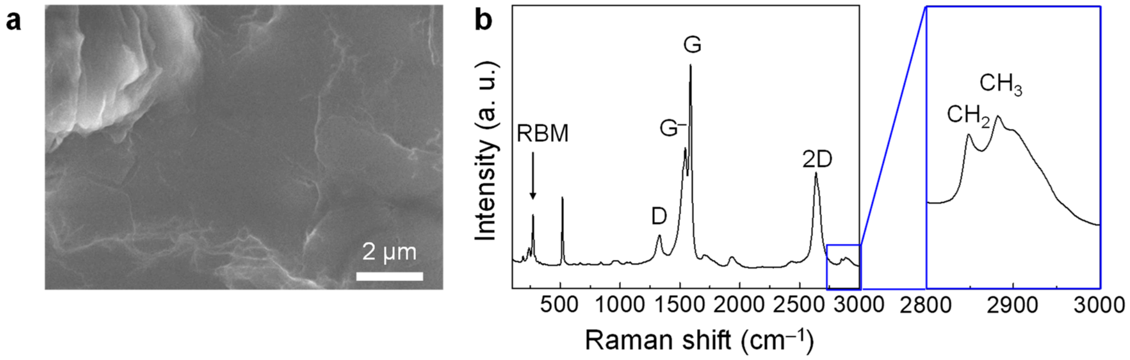

Figure S5. (a) SEM image and Raman spectrum of as-prepared non-oxidized hybrid graphene/SWNT film without removal of SDS surfactant.

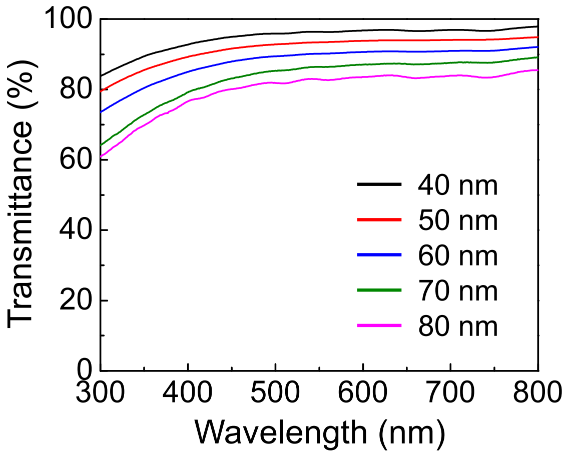

Figure S6. (a) Optical transmittance at 550 nm for 30 wt.% graphene contained hybrid electrodes with various film thickness.

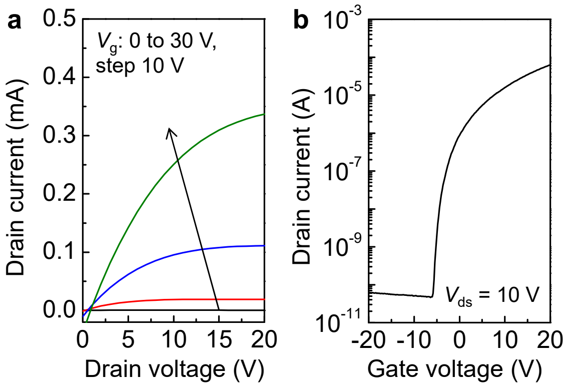

Figure S7. (a) I_{ds}-V_{ds} and (b) I_{ds}-V_g curves of IGZO TFTs with Al source and drain electrodes.
